# Creation of a Multimorbidity Health Conditions Index for Caloric Restriction Studies in Older Adults

**DOI:** 10.1093/geroni/igaf122.1021

**Published:** 2025-12-31

**Authors:** Michael Miller, Haiying Chen, Mark Espeland, Fang-Chi Hsu, Denise Houston, Anne Newman, W Jack Rejeski, Stephen Kritchevsky

**Affiliations:** Wake Forest University School of Medicine, Winston-Salem, North Carolina, United States; Wake Forest University School of Medicine, Winston-Salem, North Carolina, United States; Wake Forest University School of Medicine, Winston-Salem, North Carolina, United States; Wake Forest University School of Medicine, Winston-Salem, North Carolina, United States; Wake Forest University School of Medicine, Winston-Salem, North Carolina, United States; University of Pittsburgh, Pittsburgh, Pennsylvania, United States; Wake Forest University, Winston-Salem, North Carolina, United States; Wake Forest University School of Medicine, Winston-Salem, North Carolina, United States

## Abstract

Using multiple sources, we provide the conceptual justification and statistical support for a multimorbidity outcome associated with 29 obesity-related conditions, which we term the Health Conditions Index (HCI). The HCI was designed to capture the health effects of multi-year studies of caloric restriction in older adults with overweight or obesity. We used a subset of participants from the Health, Aging and Body Composition Cohort Study for whom weight loss would be indicated to evaluate multiple aspects of the index and its components over five years of follow-up. The 937 participants in the subset had an average age of 73 years and BMI of 30.7 kg/m2, 53% were female and 43% were Black. The components of the HCI were consistently related to initial BMI and percent body fat on cohort entry, generally showed an increasing prevalence over the 5-yr follow-up, and, as a composite index, the HCI exhibited an association between faster progression and higher initial levels of age, BMI, and percent body fat. Further, the initial HCI was associated with a statistically significant increase in the rate of mortality over 5-years of follow-up (hazard ratio [95% CI]: 1.22 [1.04, 1.44]). By using outcomes like the HCI, clinical trials of caloric restriction in older adults may gain a better understanding of how intentional weight loss relates to future risk of multiple health conditions associated with aging.

